# Passage of the Dental Bill

**Published:** 1868-04

**Authors:** 


### Editorial.

### PASSAGE OF THE DENTAL BILL.

It is with much gratification that we announce the final passage of the bill regulating the practice of Dentistry by the Legislature of the State of Ohio. A bill of this character was introduced two years ago. It has been considerably changed since that time, but we believe in every respect for the better. The law is very brief, but pertinent and comprehensive. Its execution is placed in the hands of the State Society and societies auxiliary thereto, which is certainly an improvement upon the method first contemplated.

It will be seen that the law is not operative upon any now in practice in the State of Ohio till January, 1873, at which time *all* who have not complied with its provisions will be required to do so.

But it goes into effect at once, with all those going into practice after the passage of the law, whether as beginners, or coming from any other part of the country to practice Dentistry in this State.

The State Society will convene within a few days, and make such arrangements as may be necessary for carrying out the provisions of the law. The profession in the State will be notified of these arrangements immediately upon their consummation.

We append a copy of the law.

